# Constrained statistical inference: sample-size tables for ANOVA and regression

**DOI:** 10.3389/fpsyg.2014.01565

**Published:** 2015-01-13

**Authors:** Leonard Vanbrabant, Rens Van De Schoot, Yves Rosseel

**Affiliations:** ^1^Department of Data Analysis, Ghent UniversityGhent, Belgium; ^2^Department of Methodology and Statistics, Faculty of Social and Behavioral Sciences, Utrecht UniversityUtrecht, Netherlands; ^3^Optentia Research Program, Faculty of Humanities, North-West UniversityVanderbijlpark, South Africa

**Keywords:** F-bar test statistic, inequality/order constraints, linear model, power, sample-size tables

## Abstract

Researchers in the social and behavioral sciences often have clear expectations about the order/direction of the parameters in their statistical model. For example, a researcher might expect that regression coefficient β_1_ is larger than β_2_ and β_3_. The corresponding hypothesis is *H*: β_1_ > {β_2_, β_3_} and this is known as an (order) constrained hypothesis. A major advantage of testing such a hypothesis is that power can be gained and inherently a smaller sample size is needed. This article discusses this gain in sample size reduction, when an increasing number of constraints is included into the hypothesis. The main goal is to present sample-size tables for constrained hypotheses. A sample-size table contains the necessary sample-size at a pre-specified power (say, 0.80) for an increasing number of constraints. To obtain sample-size tables, two Monte Carlo simulations were performed, one for ANOVA and one for multiple regression. Three results are salient. First, in an ANOVA the needed sample-size decreases with 30–50% when complete ordering of the parameters is taken into account. Second, small deviations from the imposed order have only a minor impact on the power. Third, at the maximum number of constraints, the linear regression results are comparable with the ANOVA results. However, in the case of fewer constraints, ordering the parameters (e.g., β_1_ > β_2_) results in a higher power than assigning a positive or a negative sign to the parameters (e.g., β_1_ > 0).

## 1. Introduction

Suppose that a group of researchers is interested in the effects of a new drug in combination with cognitive behavioral therapy (CBT) to diminish depression. One of their hypothesis is that CBT in combination with drugs is more effective than CBT only and that the new drug is more effective than the old drug. In symbols this hypothesis can be expressed as *H_CBT_*: μ_1_ < μ_2_ < μ_3_ (μ_1_ = *CBT*_*new_drug*_, μ_2_ = *CBT*_*old_drug*_, μ_3_ = *CBT*_*no_drug*_), where μ reflects the population mean for each group. To replace the old drug with the new one, the researchers want at least a medium effect size of *f* = 0.25. Classical sample-size tables based on the *F*-test (see for example Cohen, 1988) show that in case of three groups, *f* = 0.25 and a significance level of α = 0.05, 159 subjects are necessary to obtain a power of 0.80. However, the expected ordering of the means is in this case completely ignored. When the order is taken into account (here two order constraints), then the results from our simulation study (see Table 1, to be explained below) show that with fully ordered means a sample-size reduction of about 30% can be gained.

**Table 1:** **Sample-size table for ANOVA—sample size per group (*k* = 3, …, 8) at a power of 0.80 for Type J (α = 0.05), for an increasing number of *correctly specified* order constraints**.

	**Type I**	***f* = 0.10**	**0.15**	**0.20**	**0.25**	**0.30**	**0.35**	**0.40**
*n*_*k*3_0__	0.050	323	144	82	53	37	28	22
*n*_*k*3_1__	0.050	283 (−12.4%)	126	73	47 (−11.3%)	33	24	19 (−13.6%)
*n*_*k*3_2__	0.055	224 (−30.7%)	101	57	37 (−30.2%)	26	19	15 (−31.8%)
*n*_*k*4_0__	0.050	274	123	70	45	32	24	19
*n*_*k*4_1__	0.047	250 (−08.8%)	112	64	42 (−06.7%)	29	22	17 (−10.5%)
*n*_*k*4_2__	0.052	217 (−20.8%)	97	55	36 (−20.0%)	25	19	15 (−21.1%)
*n*_*k*4_3__	0.051	174 (−36.5%)	79	44	29 (−35.6%)	20	15	12 (−36.8%)
*n*_*k*5_0__	0.050	240	108	61	40	28	21	16
*n*_*k*5_1__	0.049	229 (−04.6%)	102	59	37 (−07.5%)	27	20	15 (−06.3%)
*n*_*k*5_2__	0.047	204 (−15.0%)	92	52	33 (−17.5%)	24	18	14 (−12.5%)
*n*_*k*5_3__	0.049	176 (−26.7%)	78	45	28 (−30.0%)	20	15	12 (−25.0%)
*n*_*k*5_4__	0.049	143 (−40.4%)	64	36	23 (−42.5%)	16	12	10 (−37.5%)
*n*_*k*6_0__	0.050	215	96	55	36	25	19	15
*n*_*k*6_1__	0.046	209 (−02.8%)	93	53	35 (−02.8%)	24	18	14 (−06.7%)
*n*_*k*6_2__	0.045	189 (−12.1%)	85	48	31 (−13.9%)	22	17	13 (−13.3%)
*n*_*k*6_3__	0.047	169 (−21.4%)	76	43	28 (−22.2%)	20	15	12 (−20.0%)
*n*_*k*6_4__	0.051	145 (−32.6%)	65	37	24 (−33.3%)	17	13	10 (−33.3%)
*n*_*k*6_5__	0.049	120 (−44.2%)	53	30	20 (−44.4%)	14	11	08 (−46.7%)
*n*_*k*7_0__	0.050	196	88	50	33	23	17	14
*n*_*k*7_1__	0.046	192 (−02.0%)	87	49	32 (−03.0%)	23	17	13 (−07.1%)
*n*_*k*7_2__	0.049	177 (−09.7%)	80	46	30 (−09.1%)	21	16	12 (−14.3%)
*n*_*k*7_3__	0.047	161 (−17.6%)	71	41	27 (−18.2%)	19	14	11 (−21.4%)
*n*_*k*7_4__	0.046	143 (−27.0%)	65	36	24 (−27.3%)	17	13	10 (−28.6%)
*n*_*k*7_5__	0.045	124 (−36.7%)	56	32	20 (−39.4%)	15	11	09 (−35.7%)
*n*_*k*7_6__	0.048	103 (−47.4%)	46	26	17 (−48.5%)	12	09	07 (−50.0%)
*n*_*k*8_0__	0.050	181	81	46	30	21	16	13
*n*_*k*8_1__	0.047	179 (−01.1%)	80	46	30 (−00.0%)	21	16	12 (−07.7%)
*n*_*k*8_2__	0.044	167 (−07.7%)	75	43	28 (−06.7%)	20	15	12 (−07.7%)
*n*_*k*8_3__	0.048	156 (−13.8%)	69	40	26 (−13.3%)	18	14	11 (−15.4%)
*n*_*k*8_4__	0.046	140 (−22.7%)	63	36	23 (−23.3%)	16	12	10 (−23.1%)
*n*_*k*8_5__	0.046	126 (−30.4%)	56	32	21 (−30.0%)	15	11	09 (−30.8%)
*n*_*k*8_6__	0.047	108 (−40.3%)	49	27	18 (−40.0%)	13	10	08 (−38.5%)
*n*_*k*8_7__	0.049	092 (−49.2%)	41	23	15 (−50.0%)	11	08	06 (−53.8%)

*The value between parentheses is the relative decrease in sample size*.

Consider another example of a constrained hypothesis but now in the context of linear regression. Suppose that a group of researchers wants to investigate the relation between the target variable IQ and five exploratory variables. Three exploratory variables are expected to be positively associated with an increase of IQ, while two are expected to be negatively associated:

- social skills (β_1_ > 0)
- interest in artistic activities (β_2_ > 0)
- use of complicated language patterns (β_3_ > 0)
- start walking age (β_4_ < 0)
- start talking age (β_5_ < 0)

To test this hypothesis an omnibus *F*-test is often used, where the user-specified model (including all predictors) is tested against the null model (including an intercept only). In our example, the null hypothesis is specified as *H*_0_: β_1_ = β_2_ = β_3_ = β_4_ = β_5_ = 0. Classical sample-size tables show that in case of a medium effect-size (*f*^2^ = 0.10) 135 subjects are necessary to obtain a power of 0.80 (α = 0.05). However, all information about the expected direction of the effects is completely ignored. When this information is taken into account, then our simulation results (see Table 2, to be explained below) show that with imposing five inequality constraints, a sample-size reduction of about 34% can be gained. If we impose 2 inequality constraints, the reduction drops to about 14%. This clearly shows that imposing more inequality constraints on the regression coefficients results in more power. Note that the researchers only imposed inequality constraints on the variables of interest. But, this does not have to be the case. Additional power can be gained by also assigning positive or negative associations to control variables. For example, the researchers could have controlled for socioeconomic status (SES). Although, SES is not part of the researchers main interest, they could have constrained SES to be positively associated with IQ if they have clear expectations about the sign of the effect. In this vein, *a priori* knowledge about the sign of a regression parameter can be an easy solution to increase the number of constraints and, therefore, decreasing the necessary sample-size (Hoijtink, 2012).

**Table 2:** **Sample-size table for linear regression model—total sample size at a power of 0.80 for Type J (α = 0.05) for *p* = 3, 5, 7, ρ = 0, and an increasing number of *correctly specified* inequality-constraints**.

	**Type I**	***f*^2^ = 0.02**	**0.05**	**0.08**	**0.10**	**0.15**	**0.20**	**0.25**	**0.35**
*n*_*p*3_0__	0.049	550	223	141	114	78	60	49	36
*n*_*p*3_1__	0.049	497 (−09.6%)	202	127	103 (−09.6%)	70	54	44	32 (−11.1%)
*n*_*p*3_2__	0.048	445 (−19.0%)	180	114	091 (−20.1%)	62	48	39	29 (−19.4%)
*n*_*p*3_3__	0.047	391 (−28.9%)	157	100	079 (−30.7%)	55	41	33	25 (−30.5%)
*n*_*p*5_0__	0.050	646	263	167	135	93	71	58	44
*n*_*p*5_1__	0.050	601 (−06.9%)	243	156	126 (−06.6%)	84	66	54	40 (−09.0%)
*n*_*p*5_2__	0.049	557 (−13.7%)	227	142	115 (−14.8%)	79	61	49	37 (−15.9%)
*n*_*p*5_3__	0.050	512 (−20.7%)	208	132	107 (−20.7%)	72	55	45	33 (−25.0%)
*n*_*p*5_4__	0.049	467 (−27.7%)	190	118	096 (−28.8%)	66	50	41	30 (−31.8%)
*n*_*p*5_5__	0.049	424 (−34.3%)	171	108	088 (−34.8%)	59	45	37	27 (−38.6%)
*n*_*p*7_0__	0.047	723	297	186	154	104	80	66	50
*n*_*p*7_1__	0.048	686 (−05.1%)	279	175	141 (−08.4%)	097	75	61	46 (−08.0%)
*n*_*p*7_2__	0.048	644 (−10.9%)	259	164	134 (−12.9%)	091	70	58	43 (−14.0%)
*n*_*p*7_3__	0.044	602 (−16.7%)	246	155	125 (−18.8%)	085	65	54	40 (−20.0%)
*n*_*p*7_4__	0.050	560 (−22.5%)	226	143	118 (−23.3%)	079	61	50	37 (−26.0%)
*n*_*p*7_5__	0.044	520 (−28.0%)	211	134	109 (−29.2%)	074	56	46	34 (−32.0%)
*n*_*p*7_6__	0.050	482 (−33.3%)	196	125	100 (−35.0%)	067	52	42	31 (−38.0%)
*n*_*p*7_7__	0.050	441 (−39.0%)	180	112	091 (−40.9%)	062	47	38	28 (−44.0%)

*The value between parentheses is the relative decrease in sample size*.

Constrained statistical inference (CSI) has a long history in the statistical literature. A famous work is the classical monograph by Barlow et al. (1972), which summarized the development of order CSI in the 1950s and 1960s. Robertson et al. (1988) captured the developments of CSI in the 1970s to early 1980s and Silvapulle and Sen (2005) present the state-of-the-art with respect to CSI. Although, a significant amount of new developments have taken place for the past 60 years, the relationship between power and CSI has hardly been investigated. An appealing feature of constrained hypothesis testing is that, without any additional assumptions, power can be gained (Bartholomew, 1961a,b; Perlman, 1969; Barlow et al., 1972; Robertson et al., 1988; Wolak, 1989; Silvapulle and Sen, 2005; Kuiper and Hoijtink, 2010; Kuiper et al., 2011; Van De Schoot and Strohmeier, 2011). Many applied users are familiar with this fact in the context of the classical *t*-test. Here, it is well-known that the one-sided *t*-test (e.g., μ_1_ = μ_2_ against μ_1_ > μ_2_) has more power than the two-sided *t*-test (e.g., μ_1_ = μ_2_ against μ_1_ ≠ μ_2_), because the *p*-value for the latter case has to be multiplied by two. We show that this gain in power readily extends to the setting where more than one constraint can be imposed. For example, in an ANOVA with three groups the number of order constraints may be one or two, depending on the available information about the order of the means. Hence, we present sample-size tables for constrained hypothesis tests in linear models with an increasing number of constraints. These tables will be comparable with the familiar sample-size tables in Cohen (1988) which are often seen as the “gold” standard. The major advantage of our sample-size tables is that researchers are able to look up the necessary sample size for various numbers of imposed constraints.

The remainder of this article is organized as follows. First, we introduce hypothesis test Type A and hypothesis test Type B, which are used for testing constrained hypotheses. Second, we present sample-size tables for order-constrained ANOVA, followed by sample-size tables for inequality-constrained linear regression models. For both models we present sample-size tables which depict the necessary sample size at a power of 0.80 for an increasing number of constraints. Next, we provide some guidelines for using the sample-size tables. Finally, we demonstrate the use of the sample-size tables based on the CBT and IQ examples and we provide R (R Development Core Team, 2012) code for testing the constrained hypotheses. Note that the article has been organized in such a way that the technical details are presented in the Appendices and can be skipped by less technical inclined readers who are interested primarily in the sample-size tables.

## 2. Hypothesis test type A and type B

In the statistical literature, two types of hypothesis tests are described for evaluating constrained hypotheses, namely hypothesis test Type A and Type B (Silvapulle and Sen, 2005). A formal definition of hypothesis test Type A and hypothesis test Type B is given in Supplementary Material, Appendix 1. Consider for example the following (order) constrained hypothesis: *H*: μ_1_ < μ_2_ < μ_3_. Here, the order of the means is restricted by imposing two inequality constraints. In hypothesis test Type A, the classical null hypothesis *H*_*A*0_ is tested against the (order) constrained alternative *H*_*A*1_ and can be summarized as:

Type A:

(1) $$\begin{array}{l} H_{A0}:\mu_{1}=\mu_{2}=\mu_{3}\\ H_{A1}:\mu_{1}<\mu_{2}<\mu_{3}\;. \end{array}$$

In hypothesis test Type B, the null hypothesis is the (order) constrained hypothesis *H*_*B*0_ and it is tested against the two-sided unconstrained hypothesis *H*_*B*1_ and can be summarized as:

Type B:

(2) $$\begin{array}{l} H_{B0}:\mu_{1}<\mu_{2}<\mu_{3}\\ H_{B1}:\mu_{1}\neq \mu_{2}\neq \mu_{3}\;. \end{array}$$

Note the difference with classical null hypothesis testing, where the hypothesis *H*_*A*0_ is tested against the two-sided unconstrained hypothesis *H*_*B*1_. To evaluate constrained hypotheses, like *H*: μ_1_ < μ_2_ < μ_3_, hypothesis test Type B and hypothesis test Type A are evaluated consecutively. The reason is that, if hypothesis test Type B is not rejected, then the constrained hypothesis does not fit significantly worse than the best fitting unconstrained hypothesis. In this way, hypothesis test Type B is a check for constraint misspecification. Severe violations will namely result in rejecting the constraint hypothesis (e.g., 20 < 40 < 30) and further analyses are redundant. If hypothesis test Type B is not rejected, then hypothesis test Type A is evaluated because hypothesis test Type B cannot distinguish between inequality or equality constraints. In addition, because we are mainly interested in the power of the combination of both hypothesis tests, we introduce a new hypothesis test called Type J. The power of Type J is the probability of not rejecting hypothesis test Type B times the probability that hypothesis test Type A is rejected given that hypothesis test Type B is not rejected. However, in case of constraint misspecification, we will call it pseudo power. This is because for hypothesis test Type B, power is defined as the probability that the hypothesis is correctly not rejected. Since this is not in accordance with the classical definition of power, we call it pseudo power.

In this article, we make use of the $\bar{F}$ (F-bar) statistic for testing hypothesis test Type A and hypothesis test Type B. The $\bar{F}$ is an adapted version of the well-known *F* statistic often used in ANOVA and linear regression and can deal with order/inequality constraints. The technical details of the $\bar{F}$ statistic are discussed in Supplementary Material, Appendix 2, including a brief historical overview. To calculate the *p*-value of the $\bar{F}$ statistic, we cannot rely on the null distribution of *F* as in the classical *F*-test. However, we can compute the tail probabilities of the $\bar{F}$ distribution by simulation or via the multivariate normal distribution function. The technical details for computing the *p*-value based on the two approaches are discussed in Supplementary Material, Appendix 3.

Several software routines are available for testing constrained hypotheses using the $\bar{F}$ statistic (hypothesis test Type A and Type B). Ordered means may be evaluated by the software routine “Confirmatory ANOVA” discussed in Kuiper et al. (2010). An extension for linear regression models is available in the R package ic.infer or in our own written R function csi.lm(). The function is available online at http://github.com/LeonardV/CSI_lm. Hypothesis test Type A may also be evaluated by the statistical software SAS/STAT® (SAS Institute Inc., 2012) using the PLM procedure.

## 3. Sample-size tables for order constrained ANOVA

In this section we calculate the sample size according to a power of 0.80 for hypothesis test Type J. We will in particular investigate (a) the gain in power when we impose an increasingly number of correctly specified order constraints on the One-Way ANOVA model; (b) the pseudo power when some of the means are not in line with the ordered hypothesis.

### 3.1. Correctly specified order constraints

We consider the model *y_i_* = μ_1_*x*_*i*1_ + … + μ*_k_x_ik_* + ϵ_*i*_, *i* = 1, …, *n*, where we assume that the residuals are normally distributed. Data are generated according to this model with uncorrelated independent variables, for *k* = 3, …, 8 groups, and for a variety of real differences among the population means, *f* = 0.10 (small), 0.15, 0.20, 0.25 (medium), 0.30, 0.40 (large), where *f* is defined according to Cohen (1988, pp. 274–275). We generated 20,000 datasets for *N* = 6, …, *n*, where *n* is eventually the sample-size per group at a power of 0.80. The simulated power is simply the proportion of *p*-values smaller than the pre-defined significance level. In this study we choose the arbitrary value α = 0.05. An extensive description of the simulation procedure is given in Supplementary Material, Appendix 4.

Table 1 shows the result of the simulation study in which we investigated the sample size at a power of 0.80 for different effect sizes and an increasing number of order constraints. For example, the first row (*n*_*k*3_0__) presents the sample-sizes per group for an ANOVA with *k* = 3 groups and no constraints. These sample-sizes are equal to those in Cohen (1988)^1^. The second row (*n*_*k*3_1__) shows the sample-sizes per group for *k* = 3 and 1 imposed order constraint, and so on. The values between the parentheses show the relative sample-size reduction. The second column represents the Type I error rates. The values are computed based on the smallest sample size given in the last column (*S* = 10,000, *S* is the number of datasets). All results are close to the pre-defined value of α = 0.05, despite the fact that hypothesis test Type J is a composite of hypothesis test Type A and Type B.

The results show that, for any value of *f*, the sample size decreases with the restrictiveness of the hypothesis. In other words, more information about the means, provided by the order constraints imposed on them, leads to a higher power. For example, in case of a small effect size (*f* = 0.10) and *k* = 4, the total sample size reduction with 1 constraint is 96 (274-250 = 24, 4 × 24 = 96), with 2 constraints 228 (4 × 57), and with 3 constraints 400 (4 × 100). Noteworthy, within a certain group *k* and a given number of constraints, the sample size decreases relatively equal across effect sizes. For example, if *k* = 4 and 3 constraints are imposed, the sample size decreases approximately 36%, independent of effect size. In addition, we compared the results of hypothesis test Type J with the results of hypothesis test Type A (not shown here). The results are almost identical and show only some minor fluctuations, which confirms that hypothesis test Type B only plays a significant role when the means are not in line with the imposed order.

### 3.2. Incorrect order of the means

The preceding calculations have all been for sets of means which satisfy the order constraints. Its power (read pseudo power) when the order of the means is not satisfied is also of our concern. In particular we would like to know about the power when the means are not perfectly in line with the ordered hypothesis. In this vein, we focus on the scenario that *k* = 4, *f* = 0.10, 0.25, 0.40 and three order constraints. The two outer means are fixed and only the two middle means are varied. For each value of *f* five variations are investigated according to the rule μ_*i*_γ (*i* = 2, 3), where γ = 0, −0.25, −0.50, −0.75, −1, and reflects minor to larger violations.

The results reveal that the power for Hypothesis test Type A (*H*_*A*0_ vs. *H*_*A*1_) is largely dominated by the extremes (here the first and last mean). This means that, irrespective of the deviations of the two middle means, the power is almost not affected. The results for hypothesis test Type B (*H*_*B*0_ vs. *H*_*B*1_) clearly show that the power to detect mean deviations increases with sample size. We can conclude that the pseudo power for Type J is less affected by minor mean deviations, where large violations may affect the pseudo power severely. This effect becomes more pronounced with larger effect sizes.

## 4. Sample-size tables for inequality constrained linear regression

In this section we calculate again the sample size according to a power of 0.80 for hypothesis test Type J. But now we impose only an increasing number of correctly specified inequality constraints on the regression coefficients. We consider the model *y_i_* = β_1_*x*_*i*1_ + … + β*_p_x_ip_* + ϵ_*i*_, *i* = 1, …, *n*, where we assume that the residuals are normally distributed. Data are generated according to this model with correlated independent variables and with fixed and all equal regression coefficients (β_*i*_ = 0.10). This is because in a non-experimental setting, correlated independent variables are the rule rather than the exception. Therefore, we investigate this for the situations where the predictor variables are weakly (ρ = 0.20) and strongly (ρ = 0.60) correlated. To make a fair comparison with the ANOVA results, we also take ρ = 0 into account. Let *f*^2^ be the effect size with *f*^2^ = 0.02 (small), 0.05, 0.08, 0.10 (medium), 0.15, 0.20, 0.25, 0.35 (large), where *f* is defined according to Cohen (1988, pp. 280–281). All remaining steps are identical to the ANOVA setting. A detailed description of the simulation procedure is given in Supplementary Material, Appendix 5.

The first observations that can be made on the Tables 2, 3, and 4 are that all Type I error values (see second column) are close to the pre-defined value of α = 0.05. The values are computed based on the smallest sample given in the last column. Second, in accordance with the ANOVA results, for any value of *f*^2^, the sample size decreases with the restrictiveness of the hypothesis. Third, the relative decrease is independent of effect size.

**Table 3:** **Sample-size table for linear regression model—total sample size at a power of 0.80 for Type J (α = 0.05) for *p* = 3, 5, 7, ρ = 0.20, and an increasing number of *correctly specified* inequality-constraints**.

	**Type I**	***f*^2^ = 0.02**	**0.05**	**0.08**	**0.10**	**0.15**	**0.20**	**0.25**	**0.35**
*n*_*p*3_0__	0.049	549	222	142	114	78	60	49	37
*n*_*p*3_1__	0.049	498 (−09.3%)	200	127	103 (−09.6%)	71	53	43	32 (−13.5%)
*n*_*p*3_2__	0.048	441 (−19.7%)	177	113	090 (−21.1%)	61	47	38	28 (−24.3%)
*n*_*p*3_3__	0.051	370 (−32.6%)	150	094	076 (−33.3%)	52	39	32	24 (−35.1%)
*n*_*p*5_0__	0.050	648	263	168	136	93	72	58	44
*n*_*p*5_1__	0.049	605 (−06.6%)	247	156	125 (−08.1%)	85	65	53	39 (−11.4%)
*n*_*p*5_2__	0.046	563 (−13.1%)	226	143	117 (−14.0%)	79	61	50	37 (−15.9%)
*n*_*p*5_3__	0.049	509 (−21.5%)	207	130	105 (−22.8%)	72	55	44	33 (−25.0%)
*n*_*p*5_4__	0.053	451 (−30.4%)	180	115	093 (−31.6%)	62	48	39	29 (−34.1%)
*n*_*p*5_5__	0.045	387 (−40.3%)	156	098	080 (−41.2%)	54	41	33	24 (−45.4%)
*n*_*p*7_0__	0.050	723	296	188	153	105	80	66	50
*n*_*p*7_1__	0.049	694 (−04.0%)	282	179	144 (−05.8%)	099	76	62	46 (−08.0%)
*n*_*p*7_2__	0.048	651 (−09.9%)	265	169	136 (−11.1%)	092	71	58	43 (−14.0%)
*n*_*p*7_3__	0.047	612 (−15.4%)	246	158	126 (−17.6%)	086	66	54	40 (−20.0%)
*n*_*p*7_4__	0.049	565 (−21.8%)	229	145	117 (−23.5%)	080	61	50	37 (−26.0%)
*n*_*p*7_5__	0.044	514 (−28.9%)	206	132	106 (−30.7%)	072	55	44	33 (−34.0%)
*n*_*p*7_6__	0.047	453 (−37.3%)	186	116	094 (−38.5%)	064	49	39	29 (−42.0%)
*n*_*p*7_7__	0.049	393 (−45.6%)	159	100	081 (−47.0%)	055	42	34	25 (−50.0%)

*The value between parentheses is the relative decrease in sample size*.

**Table 4:** **Sample-size table for linear regression model—total sample size at a power of 0.80 for Type J (α = 0.05) for *p* = 3, 5, 7, ρ = 0.60, and an increasing number of *correctly specified* inequality-constraints**.

	**Type I**	***f*^2^ = 0.02**	**0.05**	**0.08**	**0.10**	**0.15**	**0.20**	**0.25**	**0.35**
*n*_*p*3_0__	0.049	549	222	142	114	79	60	49	37
*n*_*p*3_1__	0.050	507 (−07.6%)	206	129	105 (−07.8%)	71	54	44	33 (−10.8%)
*n*_*p*3_2__	0.052	441 (−19.6%)	181	114	090 (−21.0%)	62	48	39	29 (−21.6%)
*n*_*p*3_3__	0.050	334 (−39.1%)	137	086	071 (−37.7%)	48	36	30	21 (−43.2%)
*n*_*p*5_0__	0.050	648	263	168	136	93	71	58	44
*n*_*p*5_1__	0.045	626 (−03.3%)	254	160	131 (−03.6%)	89	67	55	41 (−06.8%)
*n*_*p*5_2__	0.046	575 (−11.2%)	234	149	119 (−12.5%)	81	63	51	38 (−13.6%)
*n*_*p*5_3__	0.045	525 (−18.9%)	214	137	109 (−19.8%)	75	57	46	34 (−22.7%)
*n*_*p*5_4__	0.053	452 (−30.2%)	185	118	095 (−30.1%)	64	50	40	29 (−34.0%)
*n*_*p*5_5__	0.051	344 (−46.9%)	139	088	071 (−47.7%)	48	36	30	22 (−50.0%)
*n*_*p*7_0__	0.050	720	297	188	151	104	80	66	50
*n*_*p*7_1__	0.045	714 (−00.8%)	291	186	148 (−01.9%)	102	78	64	48 (−04.0%)
*n*_*p*7_2__	0.050	675 (−06.2%)	275	175	142 (−05.9%)	096	74	61	45 (−10.0%)
*n*_*p*7_3__	0.052	635 (−11.8%)	260	165	134 (−11.2%)	090	70	57	42 (−16.0%)
*n*_*p*7_4__	0.046	591 (−17.9%)	240	152	124 (−17.8%)	084	64	53	39 (−22.0%)
*n*_*p*7_5__	0.049	531 (−26.5%)	219	137	110 (−27.1%)	076	58	47	35 (−30.0%)
*n*_*p*7_6__	0.050	464 (−35.5%)	189	119	095 (−37.0%)	065	49	40	30 (−40.0%)
*n*_*p*7_7__	0.045	344 (−52.2%)	139	088	071 (−52.9%)	048	36	30	22 (−56.0%)

*The value between parentheses is the relative decrease in sample size*.

Table 2 presents the results for ρ = 0. When we compare these results with the ANOVA results in Table 1 it is clear that imposing inequality constraints (e.g., β_*i*_ > 0) on the regression coefficients leads to a lower power compared to order constraints (e.g., μ_1_ > μ_2_). For example, for the case that *k* = *p* = 5 and 4 constraints, the sample size reduction is approximately 40 and 29%, respectively. Moreover, at the maximum number of inequality constraints (here 5 constraints) the sample-size reduction of about 36% is still less than when the parameters are fully ordered. The results for a more realistic scenario (ρ = 0.20) are shown in Table 3. The findings at a maximum number of inequality constraints are comparable with the ANOVA results. For example, the total sample size decrease for *p* = 3, 5, 7 is approximately 34, 42 and 47%, respectively.

## 5. Guidelines

If researchers want to use our sample-size tables, then we recommend the following 5 steps:

Step 1: Formulate the hypothesis of interest.
Step 2a: Formulate any expectations about the order of the model parameters in terms of order constraints (i.e., means in an ANOVA setting and regression coefficients in a linear regression setting). For example, the expectation that the first mean (μ_1_) is larger than the second (μ_2_) and third mean (μ_3_) can be formulated in terms of two order constraints, namely μ_1_ > μ_2_ and μ_1_ > μ_3_.
Step 2b: Formulate any expectations about the sign of the model parameters in terms of inequality constraints. For example, the expectation that three (continuous or dummy) predictor variables are positively associated with the response variable. This can be formulated in terms of three inequality constraints, namely β_1_ > 0, β_2_ > 0 and β_3_ > 0.
Step 3: Count the number of non-redundant constraints in step 2a and/or 2b and lookup the needed sample-size in one of the sample-size tables.
Step 4: Collect the data.
Step 5: Evaluate the constrained hypothesis by using for example the csi.lm() function.

## 6. Illustrations

To illustrate our method, we consider the CBT and IQ examples. We demonstrate how to use the sample-size tables in practice and we present the R code of the csi.lm() function for testing the constrained hypotheses. The results of the analyses are also briefly discussed. The output of the csi.lm() function for the ANOVA and regression example is provided in Supplementary Materials, Appendices 6 and 7, respectively. The R code and example datasets are available online at http://github.com/LeonardV/CSI_lm.

### 6.1. ANOVA

In the introduction, we discussed the following order-constrained hypothesis (step 1):

(3) $$H_{CBT}:\mu_{new\_drug\_CBT}<\mu_{old\_drug\_CBT}<\mu_{no\_drug\_CBT},$$

where the researchers had clear expectations about the order of the three means. These expectations were translated into two order constraints between the parameters (step 2). The next step, before data collection, is to determine the necessary sample size to obtain a power of say 0.80 (α = 0.05) when the two order constraints are taken into account (step 3). Sample-size tables based on the classical *F*-test show that in case of *k* = 3 and *f* = 0.25 53 subjects per group (159 subjects in total) are necessary. If the researchers plan to use the $\bar{F}$-test instead of the classical *F*-test, then it can be retrieved from Table 1 that with two order constraints 37 subjects (111 subjects in total) are needed (see row *n*_*k*3_2__). That is a total sample-size reduction of about 48 subjects or about 30%. Then, in order to evaluate the order constrained hypothesis, using the csi.lm() function, the next four lines of R code are required (step 5):


R> data <- read.csv(“depression.csv”)
R> model <- ’depression -1 + factor
    (group)’ # -1 no intercept
R> R1 <- rbind(c(-1,1,0), c(0,-1,1))
R> fit.csi <- csi.lm(model, data, ui = R1)


In the first line the observed data are loaded into R. The data should be a data frame consisting of two columns. The first column contains the observed depression values, the second column contains the group variable. The second line is the model syntax and it is identical to the model syntax for the R function lm(). The intercept was removed from the model so that the regression coefficients correspond to the means as in an One-Way ANOVA. The third line shows the imposed order-constraints, where c(-1,1,0) indicates the first pairwise order constraint between the first and the second mean and c(0,-1,1) the second pairwise order constraint between the second and the third mean. The forth line calls the actual csi.lm() function for testing the order-constrained hypothesis. The arguments to csi.lm() are the model, the data and the matrix with the imposed constraints.

The results (see Supplementary Material, Appendix 6) show that for Hypothesis test Type B the order constrained hypothesis is not rejected in favor of the unconstrained one, $\bar{F}$_*B*_ = 0.000, *p* = 1.000 (an $\bar{F}$_*B*_-value of zero implies that the means are completely in line with the imposed order). The results for hypothesis test Type A indicate that the classical null hypothesis is rejected in favor of the constrained hypothesis, $\bar{F}$_*A*_ = 4.414, *p* = 0.038. Thus, the results are in line with the expectations of the researchers. Noteworthy, when the order is completely ignored, then the omnibus *F*-test is not significant, *F* = 1.718, *p* = 0.168. This clearly demonstrates that the $\bar{F}$-test has substantially more power than the classical *F*-test.

### 6.2. Multiple regression

The use of the linear regression sample-size tables is comparable with the ANOVA sample-size table. Recall, that in the IQ example, a group of researchers wanted to investigate the relation between the response variable IQ and five predictor variables (step 1), namely social skills (β_1_), interest in artistic activities (β_2_), use of complicated language patterns (β_3_), start walking age (β_4_), and start talking age (β_5_). Their hypothesis of interest was that the first three predictor variables are positively associated with higher levels of IQ (β_1_ > 0, β_2_ > 0 and β_3_ > 0) and that the last two predictors are negatively associated with IQ (β_4_ < 0, β_5_ < 0) (step 2). Thus, a total of five inequality constraints were imposed on the regression coefficients (step 3). Furthermore, the researchers expected a medium effect size (*f*^2^ = 0.10) for the omnibus *F*-test and a weak correlation (ρ = 0.20) among the predictor variables. All things considered, classical sample-size tables based on the *F*-test reveal that at least 136 subjects are necessary to obtain a power of 0.80 (α = 0.05). However, when the expected positive and negative associations are taken into account, then from Table 3 it can be retrieved that by means of imposing five inequality constraints, only 80 subjects are needed to maintain a power of 0.80 (see row *n*_*p*5_5__). That is a substantial sample-size reduction of about 40% or 56 subjects.

The R code to evaluate this inequality constrained hypothesis is analog to the ANOVA example (step 5):


R> data <- read.csv(“IQ.csv”)
R> model <- ’IQ social + artistic +
    language + walking + talking’
R> R1 <- rbind(c(0,1,0,0,0,0),
    c(0,0,1,0,0,0), c(0,0,0,1,0,0),
    c(0,0,0,0,-1,0), c(0,0,0,0,0,-1))
R> fit.csi <- csi.lm(model, data, ui = R1)


The results (see Supplementary Material, Appendix 7) show that the inequality constrained hypothesis is not rejected in favor of the unconstrained hypothesis, $\bar{F}$_*B*_ = 0.211, *p* = 0.847, and that the null hypothesis is rejected in favor of the constrained hypothesis, $\bar{F}$_*A*_ = 10.707, *p* = 0.019. Thus, the results are in line with the expectations of the researchers. The results for the classical *F*-test are again not significant, *F* = 2.184, *p* = 0.067.

## 7. Discussion and conclusion

In this paper we presented the results of a simulation study in which we studied the gain in power for order/inequality constrained hypotheses. The presented sample-size tables are comparable with the sample-size tables described in Cohen (1988) but with the added benefit that researchers will be able to look up the necessary sample size with a pre-defined power of 0.80 *and* number of imposed constraints.

We included an increasing number of order constraints in the One-Way ANOVA hypothesis test and inequality constraints in the linear regression hypothesis test. The ANOVA results, for *k* = 3, …, 8 groups, showed that a substantially amount of power can be gained when constraints are included in the hypothesis. Depending on the number of groups involved, a maximum sample-size reduction between 30 and 50% could be gained when the full ordering between the means is taken into account. For *k* > 4 it is questionable whether imposing less than two order constraints is sufficient for the minor gain in power; for *k* > 7 this may be questionable for less than three constraints. Furthermore, we also investigated the effect of constraint misspecification on the power. The results showed that small deviations have only a minor impact on the power.

The linear regression results reveal that, for *p* = 3, 5, 7 parameters, the power increases with the restrictiveness of the hypothesis independent of effect size. Again, a substantial power increase between approximately 30 and 50% can be gained when taking a correlation (ρ) of 0.20 between the independent variables into account. These findings are comparable with the ANOVA results, but only apply to the maximum number of constraints. In all other cases, the results showed that an ordering of the parameters leads to a higher power compared to imposing inequality constraints on the parameters. Nevertheless, full ordering of the parameters may be challenging, while imposing inequalities on the parameters may be an easier task. Hence, combining inequality constraints and order constraints may be a solution for applied users.

The current study has some limitations. In the data generating process (DGP) for the ANOVA model, we made some simplifying assumptions: the differences between the means are equally spaced, the sample size is equal in each group, there are no missing data, and the residuals are normally distributed. For the linear regression model, the DGP assumes that the correlations between the independent variables are all equal. In future research, the effects of these assumptions on a possible power drop should be studied. Moreover, we only investigated a limited set of possibilities and extensions for α = 0.01 and different power levels are desirable. However, because it is impossible to cover all possibilities, we are currently working on a user-friendly R package for constrained hypothesis testing which will include functions for sample-size and power calculations. Despite these limitations, we believe that the presented sample-size tables are a welcome addition to the applied user's toolbox, and may help convincing applied users to incorporate constraints in their hypotheses. Indeed, notwithstanding the substantial gain in power, constrained hypothesis testing is still largely unknown in the social and behavioral sciences, although the social and behavioral sciences are a good source for ordered tests. For example, in an experimental setting, the parameters of interest (e.g., means) can often be ordered easily. In a non-experimental setting variables such as “self-esteem,” “depression” or “anxiety” do not conveniently lend themselves for such ordering, but attributing a positive or a negative sign can often be done without much difficulties.

In conclusion, including prior knowledge into a hypothesis, by means of imposing constraints, results in a substantial gain in power. Researchers who are dealing with inevitable small samples in particular may benefit from this gain. Therefore, we recommend applied users to use these sample-size tables and corresponding software tools to answer their substantive research questions.

### Conflict of interest statement

The first author is a PhD fellow of the research foundation Flanders (FWO) at Ghent university (Belgium) and at Utrecht University (The Netherlands). The second author is supported by a grant from the Netherlands organization for scientific research: NWO-VENI-451-11-008.

## Footnotes

^1^The unconstrained One-Way ANOVA sample-sizes may differ slightly (±1) from the sample-sizes described in Cohen (1988). These differences can completely be attributed to the number of simulation runs

## References

[B1] Barlow R. E. Bartholomew D. J. Bremner H. M. Brunk H. D. (1972). Statistical Inference Under Order Restrictions. New York, NY: Wiley.

[B2] Bartholomew D. (1959a). A test of homogeneity for ordered alternatives. Biometrika 46, 36–48.

[B3] Bartholomew D. (1959b). A test of homogeneity for ordered alternatives. II. Biometrika 46, 328–335.

[B4] Bartholomew D. (1961a). Ordered tests in the analysis of variance. Biometrika 48, 325–332.

[B5] Bartholomew D. (1961b). A test of homogeneity of means under restricted alternatives. J. R. Stat. Soc. B 23, 239–281.

[B6] Cohen J. (1988). Statistical Power Analysis for the Behavioral Sciences, 2nd Edn. Hillsdale, NJ: Erlbaum.

[B7] Davis K. (2012). Constrained statistical inference: a hybrid of statistical theory, projective geometry and applied optimization techniques. Prog. Appl. Math. 4, 167–181.

[B8] Gouriéroux C. Holly A. Monfort A. (1982). Likelihood ratio test, wald test, and kuhn-tucker test in linear models with inequality constraints on the regression parameters. Econometrica 50, 63–80.

[B9] Grömping U. (2010). Inference with linear equality and inequality constraints using R: the package ic.infer. J. Stat. Softw. 33, 1–31.20808728

[B10] Hoijtink H. (2012). Informative Hypotheses: Theory and Practice for Behavioral and Social Scientists. Boca Raton, FL: Taylor & Francis.

[B11] Kim D. Taylor J. (1995). The restricted EM algorithm for maximum likelihood estimation under linear restrictions on the parameters. J. Am. Stat. Assoc. 90, 708–716.

[B12] Kud^o A. (1963). A multivariate analogue of the one-sided test. Biometrika 50, 403–418.

[B13] Kud^o A. Choi J. (1975). A generalized multivariate analogue of the one sided test. Mem. Facul. Sci. 29, 303–328 10.2206/kyushumfs.29.303

[B14] Kuiper R. Hoijtink H. (2010). Comparisons of means using exploratory and confirmatory approaches. Psychol. Methods 15, 69–86. 10.1037/a0018720 20230104

[B15] Kuiper R. Klugkist I. Hoijtink H. (2010). A Fortran 90 program for confirmatory analysis of variance. J. Stat. Softw. 34, 1–31.

[B16] Kuiper R. Nederhoff T. Klugkist I. (2011). Performance and Robustness of Confirmatory Approaches. Available online at: http://vkc.library.uu.nl/vkc/ms/SiteCollectionDocuments/Herbert/book%20unpublished/Kuiper,%20Nederhoff%20and%20Klugkist.pdf

[B17] Nüesch P. (1966). On the problem of testing location in multivariate populations for restricted alternatives. Ann. Math. Stat. 37, 113–119.

[B18] Perlman M. (1969). One-sided testing problems in multivariate analysis. Ann. Math. Stat. 40, 549–567.

[B19] R Development Core Team (2012). R: A Language and Environment for Statistical Computing. Vienna, Austria: R Foundation for Statistical Computing.

[B20] Robertson T. Wright F. T. Dykstra R. L. (1988). Order Restricted Statistical Inference. New York, NY: Wiley.

[B21] SAS Institute Inc (2012). SAS/STAT® 12.1 User's Guide. Cary, NC: SAS Institute Inc.

[B22] Shi N. Zheng S. Guo J. (2005). The restricted EM algorithm under inequality restrictions on the parameters. J. Multivariate Anal. 92, 53–76. 10.1016/S0047-259X(03)00134-918173855

[B23] Silvapulle M. Sen P. (2005). Constrained Statistical Inference: Order, Inequality, and Shape Constraints. Hoboken, NJ: Wiley.

[B24] Silvapulle M. (1992a). Robust tests of inequality constraints and one-sided hypotheses in the linear model. Biometrika 79, 621–630.

[B25] Silvapulle M. (1992b). Robust wald-type tests of one-sided hypotheses in the linear model. J. Am. Stat. Assoc. 87, 156–161.

[B26] Silvapulle M. (1996). On an F-type statistic for testing one-sided hypotheses and computation of chi-bar-squared weights. Stat. Probab. Lett. 28, 137–141.

[B27] Turlach B. Weingessel A. (2013). Quadprog: Functions to Solve Quadratic Programming Problems (version 1.5-5). Available online at: http://cran.r-project.org/web/packages/quadprog/quadprog.pdf

[B28] Van De Schoot R. Strohmeier D. (2011). Testing informative hypotheses in SEM increases power: an illustration contrasting classical hypothesis testing with a parametric bootstrap approach. Int. J. Behav. Dev. 35, 180–190 10.1177/0165025410397432

[B29] Van De Schoot R. Hoijtink H. Deković M. (2010). Testing inequality constrained hypotheses in SEM models. Struct. Equ. Model. 17, 443–463. 10.1080/10705511.2010.489010 24363548 PMC3868481

[B30] Wolak F. (1987). An exact test for multiple inequality and equality constraints in the linear regression model. J. Am. Stat. Assoc. 82, 782–793.

[B31] Wolak F. (1989). Testing inequality constraints in linear econometric models. J. Econom. 41, 205–235.

[B32] Yancey T. Judge G. Bock M. (1981). Testing multiple equality and inequality hypothesis is economics. Econ. Lett. 7, 249–255.

[B33] Zheng S. Shi N. Guo J. (2005). The restricted EM algorithm under linear inequalities in a linear model with missing data. Sci. China Ser. A 48, 819–828 10.1360/03ys0275

