# Supplementary material for: Constrained statistical inference: sample-size tables for ANOVA and regression
Source: Front Psychol. 2015 Jan 13;5:1565. doi: 10.3389/fpsyg.2014.01565 (PMC4292225; doi:10.3389/fpsyg.2014.01565)
Supplement: Supplementary file 1 [file DataSheet1.PDF]

1 Hypothesis test Type A and Type B

Consider the standard linear regression model,

$$\begin{aligned} y_i &= \theta_1 x_{i1} + \ldots + \theta_p x_{ip} + \epsilon_i, \ i = 1, \ldots, n. \\ &= \sum_{j=1}^{p} \theta_j x_{ij} + \epsilon_i. \end{aligned} \tag{1}$$

Hypothesis test Type A and hypothesis test Type B can be summarized as follows:

Type A:

$$\begin{array}{ll} H_{A0}: & \mathbf{R}\boldsymbol{\theta} = \mathbf{c} \\ H_{A1}: & \mathbf{R_1}\boldsymbol{\theta} \geq \mathbf{c} \ , \end{array} \tag{2}$$

Type B:

$$\begin{array}{ll} H_{B0}: & \mathbf{R_1}\boldsymbol{\theta} \geq \mathbf{c} \\ H_{B1}: & \boldsymbol{\theta} \in \mathbf{R}^p \ . \end{array} \tag{3}$$

If r is the number of inequality constraints imposed on $\boldsymbol{\theta} = (\theta_1, \ldots, \theta_p)^T$, and p the number of parameters involved, then let $\mathbf{R}$ be an $r \times p$ matrix with known constants, and $\mathbf{c}$ an $r \times 1$ vector with known constants (often this vector contains zeros). In an ANOVA, each row of matrix $\mathbf{R}$ is typically a permutation of the p-vector $(-1, 1, 0, \ldots, 0)$ and represents one pairwise constraint. In a linear regression model $\mathbf{R}$ is typically a permutation of the p-vector $(1, 0, \ldots, 0)$ and represents a one parameter constraint. Let $\mathbf{R_1}$ be a submatrix of $\mathbf{R}$ of order $q \times p$, where $q \leq r$. For example, suppose that $p = 4$ and $H_{A0}: \theta_1 = \theta_2 = \theta_3 = \theta_4$ and $H_{A1}: \theta_1 < \theta_2 < \{\theta_3, \ \theta_4\}$ (in H_{A1} no specific order between θ_3 and θ_4 is expected), then

$$\mathbf{R} = \begin{bmatrix} -1 & 1 & 0 & 0 \\ 1 & -1 & 0 & 0 \\ 0 & -1 & 1 & 0 \\ 0 & 1 & -1 & 0 \\ 0 & 0 & -1 & 1 \\ 0 & 0 & 1 & -1 \end{bmatrix} \text{ and } \mathbf{R_1} = \begin{bmatrix} -1 & 1 & 0 & 0 \\ 0 & -1 & 1 & 0 \\ 0 & -1 & 0 & 1 \end{bmatrix}.$$

Furthermore, at least one of the inequality signs in hypothesis test Type A must be a strict inequality so that the null hypothesis is not included in the constrained hypothesis.

2 The $\bar{F}$ test statistic

In the statistical literature, several approaches have been proposed for testing constrained hypotheses. Silvapulle & Sen (2005) present the state-of-the-art with respect to constrained statistical inference, see also Barlow et al. (1972) and Robertson et al. (1988). In addition to the $\bar{F}$ test statistic, some other test statistics in the framework of linear models are the E-square-bar test ($\bar{E}^2$), the Score test, the Wald test and the likelihood ratio test (LRT) (Gouriéroux et al., 1982; Silvapulle & Sen, 2005).

The $\bar{F}$ test can be calculated as follows:

$$\bar{F} = \{RSS(\boldsymbol{\theta}_{H0}) - RSS(\boldsymbol{\theta}_{H1})\}/S^2 \tag{4}$$

where $RSS(\boldsymbol{\theta})$ is the residual sum of squares under the hypothesis H and can be computed as follows:

$$RSS(\boldsymbol{\theta}) = \sum_{i=1}^{n} e_i^2, \tag{5}$$

where $e_i = (y_i - \hat{y}_i)$ and $\hat{y}_i = \hat{\theta}_1 x_{i1} + \ldots + \hat{\theta}_p x_{ip}$. This term is the main building block for the $\bar{F}$ test statistic.

In the unconstrained setting, the solution to $\hat{\boldsymbol{\theta}}$ can be obtained analytically. In case of constraints, we need to find $\tilde{\boldsymbol{\theta}}$, which is the solution to the constrained optimization problem. There are efficient computer algorithms for this optimization problem. For example, the subroutine solve.QP in the R package quadprog (Turlach & Weingessel, 2013) works well in our experience.
The $\bar{F}$ test finds its roots in Kudô (1963) who stated its null distribution, but pioneering steps were made in Bartholomew (1959a,b, 1961) which discussed the $\bar{\chi}^2$ (chi-square-bar) statistic, for the situation where the covariance matrix $\mathbf{V}$ has the form $\mathbf{V} = \sigma^2\mathbf{W}$ and is completely known. Kudô suggested the $\bar{F}$-statistic in case of k independent normal means with known covariance matrix $\mathbf{W}$ but unknown σ^2, see also Nüesch (1966). Kudô's work was extended by Kudô & Choi (1975) who generalized the result to the case when the covariance matrix is singular. This occurs when the number of imposed inequality constraints on the means exceeds the number of means involved. Yancey et al. (1981) discussed tests of the null hypothesis that a subset of the parameter vector lies in the positive orthant[1] for the special case in which the design matrix in the linear model is orthogonal. It was Wolak (1987) who generalized the results of Yancey et al. to the case of an arbitrary design matrix and general equality and inequality constraints. Silvapulle (1996) elaborated the results of Wolak for the case where the hypotheses are more general than the linear ones.

More recent developments in the context of linear models are for example inequality constrained generalized mixed models, and non-normal models such as logistic and Poisson regression, time series, and proportional hazard models (Davis, 2012). In addition, constrained robust tests have been discussed by Silvapulle (1992b,a), and Van de Schoot et al. (2010) presented a method for testing constrained hypotheses in structural equation models. The problem of constrained tests when there are missing data has been studied by Kim & Taylor (1995); Shi et al. (2005) and Zheng et al. (2005).

3 The null distribution of the $\bar{F}$ test

To compute the tail probabilities of the $\bar{F}$ statistic, we cannot rely on the null distribution of F as in the classical F test. This is because its null distribution has become a mixture of F distributions. Closed form expressions for the mixing weights for $p \leq 4$ can be found in Kudô (1963). The exact computation of the

[1]An orthant is any of the n-regions into which n-dimensional Euclidean space is divided by the coordinate planes. For example, in two dimensional space there are four orthants. The positive orthant exists of all vectors with positive coordinates.

weights for $p > 4$ is a difficult task in general. To deal with this issue, we discuss two suitable approaches. In the first approach, the p value can be computed easily and sufficiently accurately by a simulation approach. Let G denote the cumulative distribution function of the residuals where G is assumed known but σ may be unknown. For example the distribution of the residuals may be normally distributed. Then, the p value for the $\bar{F}$ statistic can be computed by using the following four steps (Silvapulle & Sen, 2005, pp. 98):

1. Generate independent observations $\{y_{ij} : i = 1, \ldots, n_j, j = 1, \ldots, p\}$ from G.
2. Compute the $\bar{F}$ statistic.
3. Repeat the previous two steps say $B = 100{,}000$ times.
4. Estimate the p value by M/B, where M is the number of times the $\bar{F}$ statistic in the second step exceeded its sample value.

Note that in the first step the observations may be generated from a distribution with any value for the mean and variance because the null distribution of the $\bar{F}$ does not depend on them, see Theorem 3.9.1 in Silvapulle & Sen (2005, pp. 97–98). The advantage of this method is that any error distribution may be used for computing the p value. The disadvantage is an increased computational cost. In the second approach, the p value may be computed economically by first simulating the mixing weights (w_i). The weight w_i is some nonnegative value and is the probability that $\tilde{\boldsymbol{\theta}}$ has exactly i positive elements. The sum of the weights from 0 to q is one. These weights explicitly depend on the covariance matrix of $\hat{\boldsymbol{\theta}}$ (Wolak, 1987). If the constrained set is the nonnegative orthant, then the weights can be computed by using the following five steps (see Silvapulle & Sen, 2005, pp. 79):

1. Generate independent observations $\{y_{ij} : i = 1, \ldots, n_j, j = 1, \ldots, p\}$ from G.
2. Compute $\tilde{\boldsymbol{\theta}}$ subject to $\boldsymbol{\theta} \geq 0$.
3. Count the number of elements of the vector $\tilde{\boldsymbol{\theta}}$ greater than zero.
4. Repeat the previous three steps say $B = 10{,}000$ times.
5. Estimate w_i by the proportion of times $\tilde{\boldsymbol{\theta}}$ has exactly i positive elements, $i = 0, \ldots, q$.

In addition, if the residuals are normally distributed, then the weights can be computed by using the multivariate normal probability distribution function. This method is implemented in the `ic.weight()` function in the `R` package ic.infer (Grömping, 2010).

Then, the p value for hypothesis test Type A can be computed as follows (Silvapulle & Sen, 2005, pp. 99):

$$\Pr(\bar{F}_A \geq \bar{f}_{A_{obs}}) = \sum_{i=0}^{q} w_i(H_0, H_1) \;\; \Pr[(r - q + i)F_{r-q+i,\nu} \geq \bar{f}_{A_{obs}}], \tag{6}$$

where ν is the error degrees of freedom and the $\bar{f}_{obs}$ is the sample value of the $\bar{F}$. For hypothesis test Type B, with only order/inequality constraints, the p value is computed as (Silvapulle & Sen, 2005, pp. 100):

$$\Pr(\bar{F}_B \geq \bar{f}_{B_{obs}}) = \sum_{i=0}^{q} w_i(H_0, H_1) \ \Pr[iF_{i,\nu} \geq \bar{f}_{B_{obs}}]. \tag{7}$$

4 Simulation 1 - correctly specified order constraints

In a one-way ANOVA, the populations differ only in their means. Let $\boldsymbol{\theta} = (\mu_1, \mu_2, \ldots, \mu_k)$ and let f be a measure of the true deviation from the null hypothesis, where f is defined according to Cohen (1988, pp. 274–275). Next, we discuss our six step simulation procedure.

In step 1, data are generated according to the model specified in Equation 1 with uncorrelated independent variables, for $k = 3, \ldots, 8$ groups and for a variety of real differences among the population means, $f = 0.10$ (small), 0.15, 0.20, 0.25 (medium), 0.30, 0.40 (large). Let the differences between the means, d, be equally spaced. Then d is defined as $d = \frac{2f\sqrt{k}}{\sqrt{\sum_{i=1}^{k}(2i-1-k)^2}}$ under the restriction that $\sum_{i=1}^{k} \mu_i = 0$ and $\sigma = 1$. The smallest mean, μ_1, is determined by $\mu_1 = \frac{-(k-1)d}{2}$. For example, if $k = 4$ and the effect size $f = 0.25$, then $d = \sqrt{\frac{1}{20}}$ and $\mu_1 = -0.335$. Then, $\mu_2 = \mu_1 + d$, $\mu_3 = \mu_1 + 2d$ and $\mu_4 = \mu_1 + 3d$.

In step 2, we generate $S = 20,000$ datasets according to the data generating process described in step 1 for $N = 6, \ldots, n$, where n is eventually the sample size per group at a power of 0.80.

In step 3, we fit the equality-constrained model (H_{A0}), the order-constrained model (H_{A1}) and the two-sided unconstrained model (H_{B1}) and calculate for each model the RSS_H. The imposed order constraints are of the form H: $\mu_t - \mu_s \geq 0$.

In step 4, we calculate the $\bar{F}$ values for hypothesis test Type A and Type B according to Equation 4.

Then, in step 5, we compute the p-value for hypothesis test Type A and hypothesis test Type B. This is done according to Equation 6 for hypothesis test Type A and according to Equations 7 for hypothesis test Type B, which are provided in Appendix 3.

Finally in step 6, we calculate the power for hypothesis tests Type A, Type B, and Type J. The power is simply the proportion of p-values smaller than the predefined significance level. In this study we choose the arbitrary value $\alpha = 0.05$. The conditional power is computed by $\hat{P}(\bar{b}) \times \hat{P}(a|\bar{b})$, where $\hat{P}(a)$ is the proportion of significant results for hypothesis test Type A, and $\hat{P}(\bar{b})$ is the proportion of non-significant results for hypothesis test Type B.

5 Simulation 2 - correctly specified inequality constraints

In a linear regression analysis, let $\boldsymbol{\theta} = (\beta_1, \beta_2, \ldots, \beta_p)^T$ and let $f^2 = \frac{R^2}{1-R^2}$, where R^2 is the determination coefficient. Again, a six step simulation procedure is used, similar as for the ANOVA setting.
In step 1, data are generated according to Equation 1 with fixed and all equal parameters ($\beta_i = 0.10$). Let f^2 indicate the effect size with $f^2 = 0.02$ (small), 0.05, 0.08, 0.10 (medium), 0.15, 0.20, 0.25, 0.35 (large). Since we hold the parameters fixed, generating data for a predefined R^2 boils down to determining σ^2, where $\sigma^2 = (\boldsymbol{\theta}^\top \boldsymbol{\Sigma}_{\boldsymbol{X}} \boldsymbol{\theta})(1 - R^2)/R^2$ and $\boldsymbol{\Sigma}_{\boldsymbol{X}}$ is the covariance-matrix for the covariances between the independent variables. We take this latter into account because in a non-experimental setting, correlated independent variables are the rule rather than the exception. Therefore, we investigate this for the situations where $\boldsymbol{\Sigma}_{\mathbf{X}}$ is a compound symmetry matrix with ones on the diagonal and values of ρ ($\rho = 0$, 0.20 and 0.60) elsewhere. We take the value $\rho = 0$ into account to make a fair comparison with the ANOVA model. Furthermore, in this study we will limit ourselves to $p = 3$, 5, and 7 variables.
In step 2, we generate $S = 20,000$ datasets according to the data generating process described in step 1 for $N = 6, \ldots, n$, where n is the total sample size at a power of 0.80.
Step 3 corresponds to the ANOVA setting with the exception that we impose an increasing number of correctly specified inequality constraints of the form H: $\beta_i \geq 0$ on the model.
Step 4, 5 and 6 are again identical to the ANOVA setting.

6 Output of the csi() function for the CBT example

```
#observed test statistics for hypothesis test Type A and Type B
$T.obs
 Fbar.A  Fbar.B
4.41444 0.00000

#number of active constraints
$iact
[1] 0

$p.value
    Fbar.A     Fbar.B
0.03813885 1.00000000

$Rboot.tot
NULL

#matrix R1, where each row reflects a pairwise constraint
$ui
     [,1] [,2] [,3]
```

```
[1,]   -1    1    0
[2,]    0   -1    1

#number of equality constraints
$meq
[1] 0

#mixing weights
$wt.bar
        2         1         0
0.1666667 0.5000000 0.3333333

#constrained R-square
$R2
[1] 0.04555432

#parameter estimates under HA0
$par.h0
[1] 0.8752613 0.8752613 0.8752613

#parameter estimates under HA1/HB0
$par.h1
[1] -1.7856216  0.9637027  3.4477027

#parameter estimates under HB1
$par.h2
[1] -1.7856216  0.9637027  3.4477027
```

7 Output of the csi() function for the IQ example

```
#observed test statistics for hypothesis test Type A and Type B
$T.obs
    Fbar.A     Fbar.B
10.7070949  0.2109297

#number of active constraints
$iact
[1] 5

$p.value
    Fbar.A     Fbar.B
0.01935583 0.84701892

$Rboot.tot
NULL

#matrix R1, where each row reflects a pairwise constraint
```

```
$ui
     [,1] [,2] [,3] [,4] [,5] [,6]
[1,]    0    1    0    0    0    0
[2,]    0    0    1    0    0    0
[3,]    0    0    0    1    0    0
[4,]    0    0    0    0   -1    0
[5,]    0    0    0    0    0   -1

#number of equality constraints
$meq
[1] 0

#mixing weights
$wt.bar
         5          4          3          2          1          0
0.04246308 0.19426909 0.34338421 0.29141320 0.11415272 0.01431771

#constrained R-square
$R2
[1] 0.141035

#parameter estimates under HA0
$par.h0
[1] -0.09561598 -0.09561598 -0.09561598 -0.09561598 -0.09561598 -0.09561598

#parameter estimates under HA1/HB0
$par.h1
[1] -0.09561598  0.15665820  0.13335288  0.33557004 -0.10531778  0.00000000

#parameter estimates under HB1
$par.h2
[1] -0.09561598  0.13601500  0.11270968  0.31492684 -0.12596098  0.09633493
```
